# Supplementary material for: Potent acridone antimalarial against all three life stages of Plasmodium
Source: Nat Commun. 2026 Apr 14;17:4230. doi: 10.1038/s41467-026-71708-1 (PMC13168289; doi:10.1038/s41467-026-71708-1)
Supplement: Supplementary file 1 — Supplementary Information [file 41467_2026_71708_MOESM1_ESM.pdf]

Supplementary Information

This file contains Supplementary Tables 1–6, Supplementary Figures 1–14, Supplementary Notes and Supplementary Methods.

Supplementary Table 1. In vitro cytotoxicity of T111 and reference antimalarials against human HepG2 cells.

drug	inhibition IC_{50} (nM)[a] vs HepG2
T111	>200,000
chloroquine	68,645 ± 19,837
atovaquone	61,196 ± 17,687
mefloquine	4,935 ± 799

[a]IC_{50} values represent the mean ± SEM derived from n=3 independent biological replicates, each performed in technical duplicate.

Supplementary Table 2. In vitro hERG inhibitory activity of T111.

drug	concentration (μM)	%inhibition[a]
T111	3.125	2.81
	6.25	5.29
	12.5	3.73
	25	6.91
	50	1.87
	100	2.37
verapamil[b]	0.01	7.85
	0.03	10.5
	0.1	26.3
	0.3	46.8
	1	69.8
	3	91.8
0.33% DMSO	-	6.75

[a]values represent the mean of n=2 technical replicates performed using a hERG-expressing CHO-K1 cell line.
[b]verapamil (IC_{50} = 0.334 μM) was used as a positive reference control to validate assay sensitivity.

Supplementary Table 3. Toxicokinetic parameters of T111 in rats following single and repeated oral administration.

drug dose (mg/kg)	Day 1[a, b]						Day 7[a, c]					
	male			female			male			female		
	25	100	400	25	100	400	25	100	400	25	100	400
$t_{1/2}$ (h)	NC[d]	NC	NC	NC	NC	NC	21	25	18	13.5	14.5	15
T_{max} (h)	8	8	8	8	24	1	8	2	8	8	2	8
C_{max} (ng/mL)	499	592	367	439	532	365	499	676	507	338	1,070	847
AUC_{last} (h.ng/mL)	9,300	11,100	5,340	8,090	10,700	4,400	9,570	17,700	14,900	11,100	29,900	16,500
AUC_{inf} (h.ng/mL)	NC	NC	NC	NC	NC	NC	6,400	19,100	15,000	11,100	30,300	16,600
Cl/F (mL/h/kg)	NC	NC	NC	NC	NC	NC	2,780	5,960	33,300	2,870	4,260	31,600

[a]values represent the mean toxicokinetic parameters derived from n=3 independent biological replicates (individual rats) per sex and dose group.
[b]Day 1 parameters were determined following a single oral dose (0.5–24 h sampling).
[c]Day 7 parameters were determined following seven consecutive daily doses (0.5–96 h sampling).
[d]NC: not calculated. $t_{1/2}$, AUC_{inf}, and Cl/F on Day 1 could not be calculated due to insufficient data points in the terminal phase of the plasma concentration-time profile.
$t_{1/2}$, terminal half-life; T_{max}, time to maximum concentration; C_{max}, maximum observed concentration; AUC_{last}, area under the curve to the last measurable time point; AUC_{inf}, area under the curve extrapolated to infinity; Cl/F, apparent oral clearance.

Supplementary Table 4. In vitro activity of clinical antimalarials against T111-resistant *P. falciparum* mutant lines.

parasite line	standard antimalarials			
	CQ (IC_{50}, nM)[a]	PIP (IC_{50}, nM)[a]	LUM (IC_{50}, nM)[a]	DHA (IC_{50}, nM)[a]
Dd2	181 ± 15	7.4 ± 0.57	1.2 ± 0.098	1.7 ± 0.14
Dd2-A$^{131S\text{-}140I\text{-}259L\text{-}264L}$	205 ± 21	9.9 ± 0.65	1.7 ± 0.086	2.3 ± 0.25
Dd2-B$^{131S\text{-}140I\text{-}259L\text{-}264L}$	168 ± 31	12 ± 2.3	1.7 ± 0.36	2.3 ± 0.18
Dd2-$D_0^{119L\text{-}131S\text{-}259L}$‡	210 ± 34	11 ± 1.2	1.5 ± 0.11	2.2 ± 0.18
Dd2-$D_1^{131S\text{-}140I\text{-}259L}$‡	162 ± 18	9.1 ± 1.7	2.4 ± 0.11	2.4 ± 0.20

[a]IC_{50} values represent the mean ± SEM of n≥ 3 independent biological replicates.
‡parasite lines harboring a mutation in *pfdhodh.*
CQ, chloroquine; PIP, piperaquine; LUM, lumefantrine; DHA, dihydroartemisinin.

Supplementary Table 5. Whole-genome sequencing (WGS) analysis of T111-resistant *P. falciparum* mutants.[a]

gene	description	Flask A<br>400 nM<br>66 weeks	Flask A<br>1000 nM<br>77 weeks	Flask B<br>400 nM<br>65 weeks	Flask B<br>1000 nM<br>73 weeks	Flask C0<br>600 nM<br>> 39 weeks	Flask C1<br>600 nM<br>> 39 weeks
PfDd2_010007000	nucleoside transporter 4	c.368C>T<br>p.Ser123Phe					
PfDd2_020022700	liver stage antigen 3		c.1949G>C<br>p.Ser650Thr		c.1949G>C<br>p.Ser650Thr		
PfDd2_030014300	N2227-like protein, putative						c.1660T>A<br>p.Cys554Ser
PfDd2_040009300	SET domain protein, putative					c.3467C>G<br>p.Pro1156Arg	c.3467C>G<br>p.Pro1156Arg
PfDd2_050027900	multidrug resistance protein 1	c.257T>A<br>p.Phe86Tyr					
PfDd2_060008000	dihydroorotate dehydrogenase					c.827G>T<br>p.Cys276Phe	c.827G>T<br>p.Cys276Phe
PfDd2_060017000	eukaryotic translation initiation factor 3 subunit L, putative					c.1235G>A<br>p.Ser412Asn	
PfDd2_060020400	cdc2-related protein kinase 5					c.1000G>A<br>p.Asp334Asn	
PfDd2_060035000	DNA polymerase epsilon catalytic subunit A, putative					c.7402G>A<br>p.Glu2468Lys	c.7402G>A<br>p.Glu2468Lys
PfDd2_080032700	conserved Plasmodium protein, unknown function			c.3214C>A<br>p.His1072Asn			
PfDd2_090022100	conserved Plasmodium protein, unknown function				c.2984A>G<br>p.Asn995Ser		
PfDd2_100013400	histone deacetylase 2	c.3233A>G<br>p.Asp1078Gly					
PfDd2_100024100	conserved Plasmodium protein, unknown function					c.317G>A<br>p.Cys106Tyr	c.317G>A<br>p.Cys106Tyr
PfDd2_100043400	gametocyte-specific protein					c.11473G>A<br>p.Val3825Ile	
PfDd2_110034900	conserved Plasmodium protein, unknown function					c.4274A>T<br>p.Asn1425Ile	c.4274A>T<br>p.Asn1425Ile
PfDd2_120046200	erythrocyte membrane protein 1, PfEMP1	c.688G>A<br>p.Glu230Lys	c.688G>A<br>p.Glu230Lys				c.678A>T<br>p.Glu226Asp
PfDd2_140034700	acid phosphatase, putative	c.3153A>T<br>p.Lys1051Asn	c.3153A>T<br>p.Lys1051Asn		c.3153A>T<br>p.Lys1051Asn		
PfDd2_000011300	cytochrome b	c.391G>A<br>p.Gly131Ser<br>c.418G>A<br>p.Val140Leu<br>c.775G>T<br>p.Val259Leu	c.391G>A<br>p.Gly131Ser<br>c.418G>A<br>p.Val140Leu<br>c.775G>T<br>p.Val259Leu<br>c.790T>C<br>p.Phe264Leu	c.259T>G<br>p.Leu87Val<br>c.391G>A<br>p.Gly131Ser<br>c.418G>A<br>p.Val140Leu<br>c.775G>T<br>p.Val259Leu	c.391G>A<br>p.Gly131Ser<br>c.418G>A<br>p.Val140Leu<br>c.775G>T<br>p.Val259Leu<br>c.790T>C<br>p.Phe264Leu	c.355A>C<br>p.Ile119Leu<br>c.391G>A<br>p.Gly131Ser<br>c.775G>T<br>p.Val259Leu	c.391G>A<br>p.Gly131Ser<br>c.418G>A<br>p.Val140Leu<br>c.775G>T<br>p.Val259Leu

[a]mutations are reported for cloned parasites isolated from the denoted flasks and selection parameters; only mutations present at an odds ratio >100 compared to the parental Dd2 strain are shown.

Supplementary Table 6. In vivo efficacy of T111 against ATV- and ELQ-300-resistant *P. yoelii* mutant lines.

	ED_{50} (mg/kg/day)[a]		
P. yoelii strain	ATV	ELQ-300	T111
wild type	0.064 (0.049–0.085)	0.031 (0.029–0.033)	0.24 (0.20–0.29)
ATV^{r}_{Y268S}	15.3 (9.9–24)	0.040 (0.036–0.044)	0.31 (0.29–0.34)
$ELQ\text{-}300^{r}_{I22L}$	0.068 (0.053–0.087)	1.27 (0.97–1.67)	0.25 (0.21–0.30)

[a]ED_{50} values represent the mean values derived from non-linear regression analysis of the 4-day suppression model using four- to five-week-old CF1 mice (n=4 per group). Values in parentheses represent the corresponding 95% Confidence Intervals (CI).

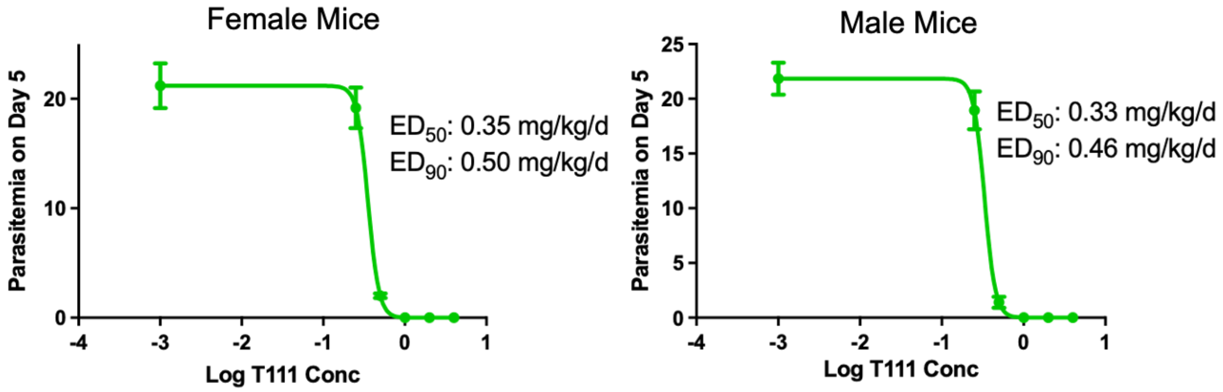

Supplementary Figure 1. T111 oral efficacy in female and male mice. Comparison of in vivo efficacy against blood-stage *P. yoelii* in female and male 4–5-week-old CF1 mice using a 4-day suppression model. Center values represent the mean, and error bars represent the SEM. Results are derived from n=4 independent biological replicates (individual mice) per sex, per treatment group. Effective doses for 50% (ED_{50}) and 90% (ED_{90}) reduction in parasitemia are indicated for each sex.

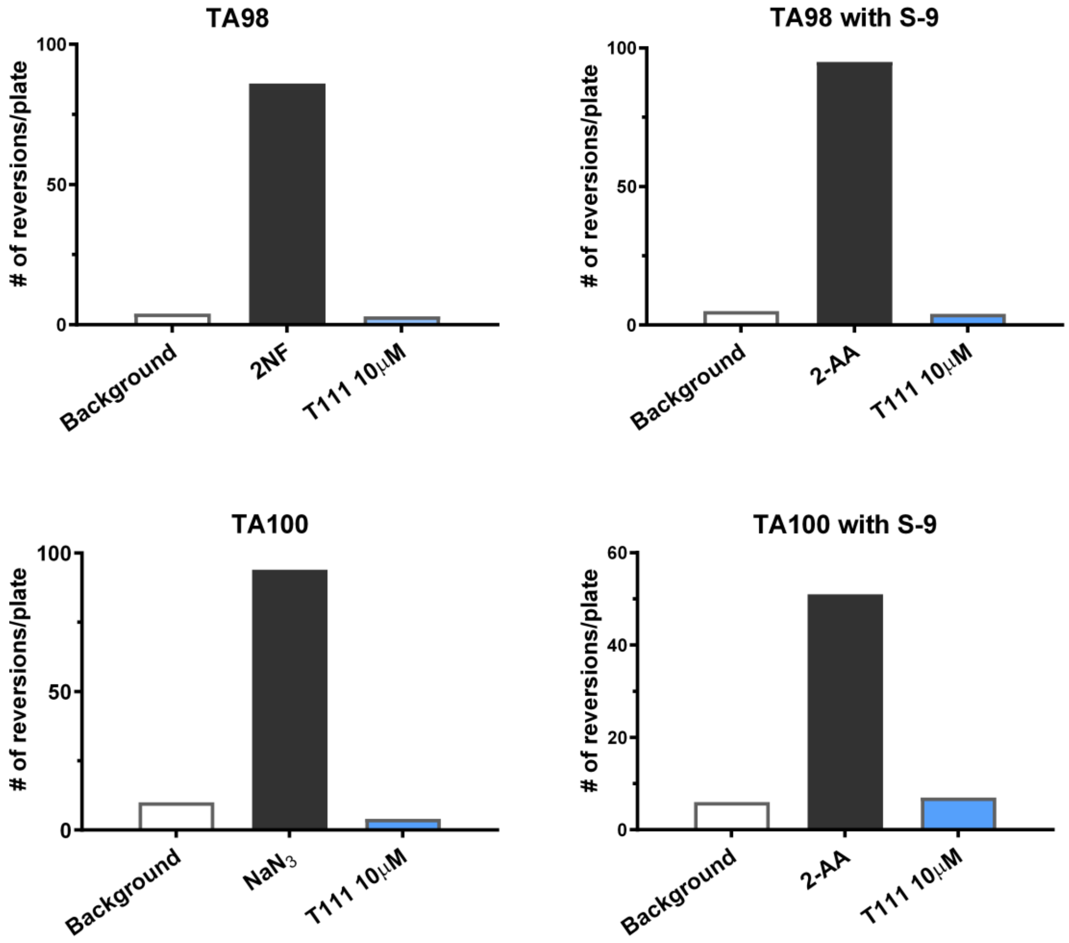

Supplementary Figure 2. **In vitro mutagenicity screening of T111.** Evaluation of mutagenic potential using *Salmonella typhimurium* strains TA98 and TA100, both with and without S9 metabolic activation. Data represent the number of revertant colonies (reversions) per plate. Results are derived from n=96 independent biological replicates (individual wells within a 96-well plate format) per condition. Positive controls include 2-nitrofluorene (2NF), 2-aminoanthracene (2-AA), and sodium azide (NaN_3). Background represents the spontaneous reversion frequency.

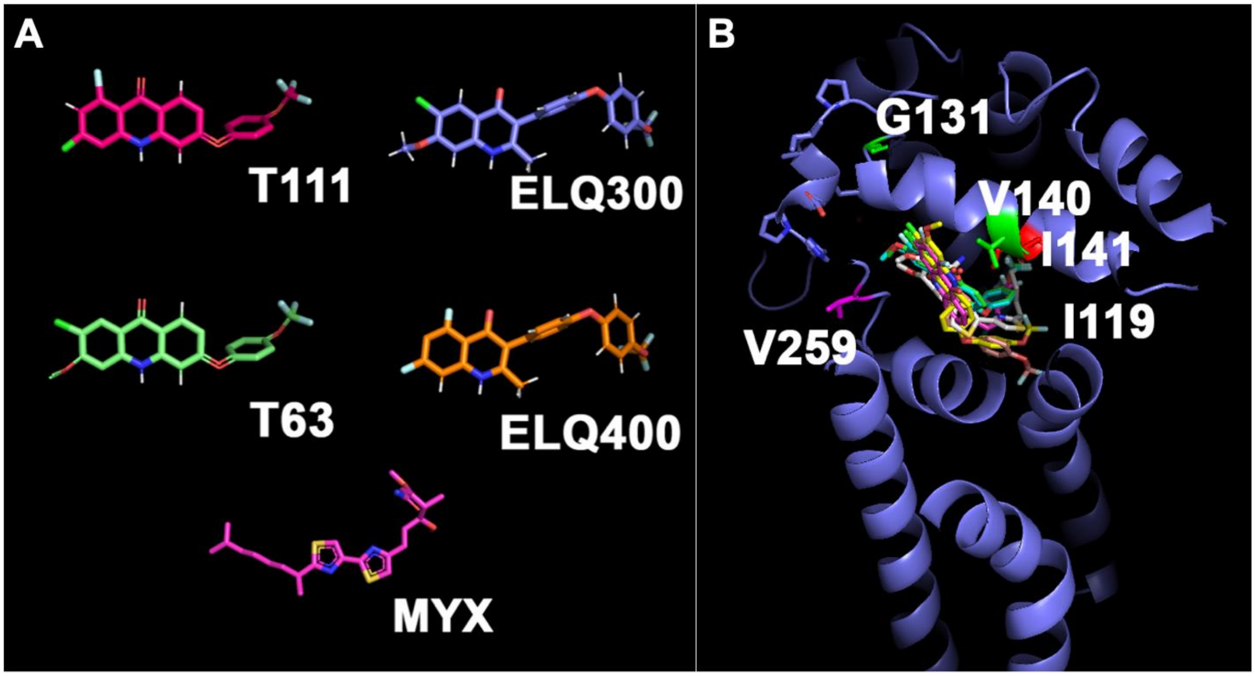

Supplementary Figure 3. Molecular docking of T111 and reference inhibitors into the *P. falciparum* CytB Q_o site. (A) Molecular structures of *cytochrome b* (*cyt b*) inhibitors. Structures are shown for T111 (pink), T63 (green), ELQ300 (blue), ELQ400 (orange), and myxothiazol (MYX, magenta). **(B)** Molecular docking models of inhibitors binding to the *P. falciparum* CytB complex. The structural overlay shows the predicted binding orientations of the inhibitors within the Q_o site. Relevant Q_o residues associated with resistance mutations (I119, G131, V140, I141, and V259) are labeled and displayed as sticks. Computational docking was performed using AutoDock 4.0. Estimated free energy of binding (ΔG_{bind}) and inhibition constants (K_i) for these ligands are provided in the Source Data file.

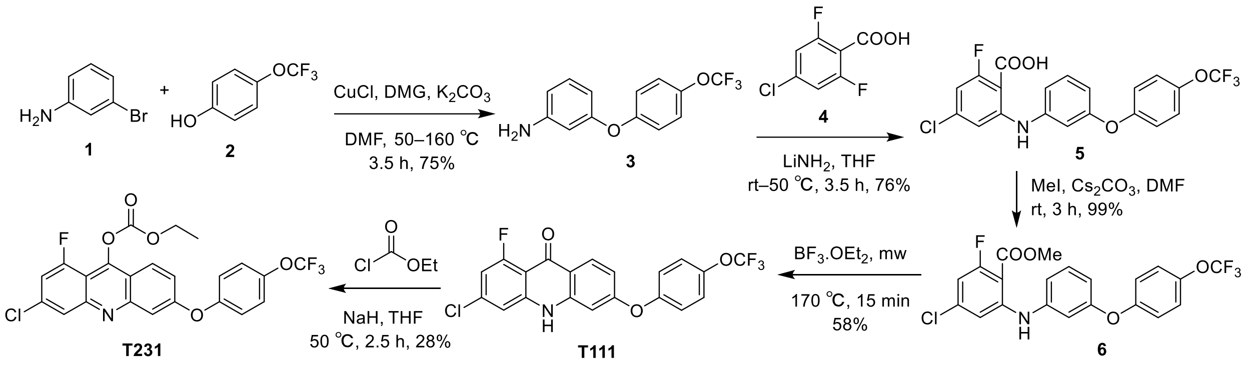

Supplementary Figure 4. Synthetic route for T111 and its carbonate prodrug T231. Chemical synthesis of the lead acridone T111 and the ethyl carbonate prodrug T231. Chemical structures and schemes were generated using ChemDraw (version 23.1.1). DMG, N,N-dimethylglycine; DMF, dimethylformamide; THF, tetrahydrofuran; MeI, methyl iodide; rt, room temperature; mw, microwave.

Supplementary Notes. NMR spectra were recorded on a Bruker AMX-400 spectrometer at 400 MHz. NMR experiments were recorded in $CDCl_3$ and DMSO-d_6 at 25 °C. Chemical shifts (δ) are given in parts per million (ppm) downfield from internal standard tetramethylsilane (TMS), and coupling constants (J) are expressed in Hertz (Hz). NMR data were processed and analyzed using TopSpin (version 4.4.1). High-resolution mass spectra (HRMS) (electrospray ionization (ESI)) were recorded on a vanquish UHPLC/HPLC system coupled with a high resolution (35,000) Q Exactive Orbitrap mass spectrometer. HRMS data were processed and analyzed using Thermo Xcalibur Qual Browser (version 4.1.50). GC-MS was performed using an Agilent Technologies 7890B gas chromatograph (30 m, DBS column set at either 100 or 200 ℃ for 2 min then at 30 ℃/min ramp to 300 ℃ with inlet temperature set at 250 ℃) using an Agilent Technologies 5977A mass-selective detector operating at 70 eV. Flash chromatography on silica gel was performed on either Isolera One flash chromatography system from Biotage or CombiFlash instruments with hexanes and ethyl acetate as eluents. Unless otherwise stated, all reagents and solvents were purchased from commercial suppliers and used without further purification. Reactions that required anhydrous conditions were carried out under an atmosphere of argon. The microwave reactions were conducted using Biotage® Initiator+ microwave synthesizer. Analytical HPLC analysis was performed on an Agilent 1260 Infinity II LC System using C8 column (2.1 mm × 50 mm) with a linear elution gradient of water/methanol (containing 10 mM ammonium acetate) ranging from 50:50 to 0:100 for 10 min at a flow rate of 0.5 mL/min, at 254 nm. A purity of >99.5% has been established for both T111 and its prodrug, T231.

Supplementary Methods

Synthesis of 3-(4-(Trifluoromethoxy)phenoxy)aniline (3). A mixture of copper (I) chloride (CuCl) (5.76 g, 58.2 mmol) and *N*,*N*-dimethylglycine (DMG) (3.99 g, 38.7 mmol) in 100 mL of dimethylformamide (DMF) was stirred under vacuum at 50 °C for 15 min. To the blue-colored catalyst mixture were added anhydrous K_2CO_3 (100.4 g, 727 mmol), *m*-bromoaniline (**1**) (50.0 g, 291 mmol), 4-(trifluoromethoxy)phenol (**2**) (51.7 g, 291 mmol), and 500 mL of DMF. The reaction mixture was degassed again at 50 °C for 15 min, purged with argon, then stirred at 160 °C for 3 h. Upon cooling to room temperature, the reaction mixture was filtered through a pad of silica gel and washed with ethyl acetate. The filtrate was concentrated by rotary evaporator and the crude product was chromatographed on silica gel, with hexanes/ethyl acetate as eluent, to afford the title compound **3** as a light brown oil (59.0 g, 75%). The identity of **3** was further confirmed by GC-MS analysis and characterized by ^{1}H NMR (Supplementary Figure 5) and HRMS (Supplementary Figure 6) spectral data. ^{1}H NMR ($CDCl_3$, 400 MHz): δ 7.10 (d, J = 8.9 Hz, 4H), 6.84–6.81 (m, 4H), 5.89–5.52 (m, 2H); HRMS (ESI) *m/z*: $[M + H]^+$ Calcd for $C_{13}H_{11}F_3NO_2$ 270.0736; Found 270.0733.

Synthesis of 4-Chloro-2-fluoro-6-((3-(4-(trifluoromethoxy)phenoxy)phenyl)amino)benzoic acid (5). A solution of $LiNH_2$ (22.0 g, 959 mmol) in 300 mL anhydrous tetrahydrofuran (THF) was stirred for 15 min at room temperature, followed by slow addition of a slurry of **3** (73.7 g, 274 mmol) and 4-chloro-2,6-difluorobenzoic acid (**4**) (52.8 g, 274 mmol) in THF (200 mL). The mixture was stirred for 1.5 h at 50 °C, when sudden bubbling was observed and the slurry turned into a clear solution. After an additional 1.5 h the completion of the reaction was confirmed by GC-MS analysis. Acetonitrile (100 mL) was added to the reaction mixture and stirred for 1 h, then the solvents were removed under reduced pressure. Addition of 200 mL ethanol and 200 mL water, followed by acidification with 2 N HCl yielded a precipitate that was filtered and washed with water. The crude solid material was chromatographed on silica gel, with hexanes/ethyl acetate as eluent, to afford the title compound **5** as a beige solid (92.2 g, 76%). The identity of **5** was further confirmed by GC-MS analysis and characterized by ^{1}H NMR (Supplementary Figure 7) and HRMS (Supplementary Figure 8) spectral data. ^{1}H NMR (DMSO-d_6, 400 MHz): δ 13.7 (br s, 1H), 9.16 (s, 1H), 7.39 (d, J = 8.2 Hz, 2H), 7.37 (d, J = 8.1 Hz, 1H), 7.20–7.15 (m, 2H), 7.03 (ddd, J = 8.1, 2.1, 0.8 Hz, 1H), 6.95 (dd, J = 1.4, 0.4 Hz, 1H), 6.88 (t, J = 2.2 Hz, 1H), 6.84 (dd, J = 10.6,

1.9 Hz, 1H), 6.77 (ddd, J = 8.2, 2.4, 0.8 Hz, 1H); HRMS (ESI) m/z: $[M + H]^+$ Calcd for $C_{20}H_{13}ClF_4NO_4$ 442.0464; Found 442.0464.

Synthesis of Methyl 4-Chloro-2-fluoro-6-((3-(4-(trifluoromethoxy)phenoxy)phenyl)amino)benzoate (6). To a stirred solution of **5** (41.0 g, 92.8 mmol) in DMF (300 mL) was added Cs_2CO_3 (16.6 g, 51.0 mmol), followed by slow addition of methyl iodide (MeI) (13.8 g, 97.4 mmol). The mixture was stirred at room temperature for 3 h, and the consumption of **5** was confirmed by GC-MS analysis. The solid residue was then filtered off, and washed with methanol. The filtrate was concentrated under reduced pressure to afford the crude product, which was chromatographed on silica gel with hexanes/ethyl acetate as eluent, to yield **6** as a white solid (41.8 g, 99%). Compound **6** was characterized by ^{1}H NMR (Supplementary Figure 9) and HRMS (Supplementary Figure 10) spectral data. ^{1}H NMR (DMSO-d_6, 400 MHz): δ 8.81 (s, 1H), 7.41–7.35 (m, 3H), 7.19–7.16 (m, 2H), 7.00–6.97 (m, 2H), 6.90 (dd, J = 10.5, 1.8 Hz, 1H), 6.83 (t, J = 2.2 Hz, 1H), 6.77 (dd, J = 8.1, 1.8 Hz, 1H), 3.80 (s, 3H); HRMS (ESI) m/z: $[M + H]^+$ Calcd for $C_{21}H_{15}ClF_4NO_4$ 456.0620; Found 456.0613

Synthesis of Acridone Analog T111. To a microwave reaction vial were added compound **6** (6.00 g, 13.2 mmol) and $BF_3.Et_2O$ (8.14 mL, 65.9 mmol), and the mixture was exposed to microwave (mw) radiation for 15 min at 170 °C. After cooling to room temperature, the reaction mixture was poured into water (100 mL) and allowed to stir for 5 min. The solid material was filtered by a sintered funnel and washed with water, then dried. Recrystallization from DMF/ethanol (3:1) yielded T111 as a white solid (3.23 g, 58%). Due to the limited volume capacity of the microwave reaction vials, we conducted multiple 6.00 g scale batches to generate over 50 g of the T111. T111 was characterized by ^{1}H NMR (Supplementary Figure 11) and HRMS (Supplementary Figure 12) spectral data. ^{1}H NMR (400 MHz; DMSO-d_6): δ 11.74 (s, 1H), 8.17 (d, J = 8.9 Hz, 1H), 7.52 (d, J = 8.4 Hz, 2H), 7.37 (m, 2H), 7.21 (s, 1H), 7.09 (dd, J = 11.5, 1.8 Hz, 1H), 7.00 (dd, J = 8.9, 2.3 Hz, 1H), 6.76 (d, J = 2.3 Hz, 1H); HRMS (ESI) m/z: $[M + H]^+$ Calcd for $C_{20}H_{11}ClF_4NO_3$ 424.0358; Found 424.0350.

Synthesis of Prodrug T231. A slurry of T111 (1.27 g, 3.00 mmol) in 50 mL of THF was added NaH (0.215 g, 9.00 mmol) under argon, and stirred at 50 °C for 15 min. To the reaction mixture was added ethyl chloroformate (0.650 g, 6.00 mmol), and stirred at 50 °C for 2 h. After quenching with isopropanol and methanol, the crude reaction mixture was concentrated, and flash chromatography with hexanes/ethyl acetate as eluent yielded T231 as a white solid (0.413 g, 28%).

T231 was characterized by ^{1}H NMR (Supplementary Figure 13) and HRMS (Supplementary Figure 14) spectral data. ^{1}H NMR (400 MHz; DMSO-d_6): δ 8.30 (d, J = 9.4 Hz, 1H), 8.04 (s, 1H), 7.68–7.62 (m, 2H), 7.54 (d, J = 8.6 Hz, 2H), 7.44 (d, J = 9.0 Hz, 2H), 7.29 (d, J = 2.1 Hz, 1H), 4.41 (q, J = 7.0 Hz, 2H), 1.37 (t, J = 7.0 Hz, 3H); HRMS (ESI) *m/z*: $[M + H]^+$ Calcd for $C_{23}H_{15}ClF_4NO_5$ 496.0569; Found 496.0573.

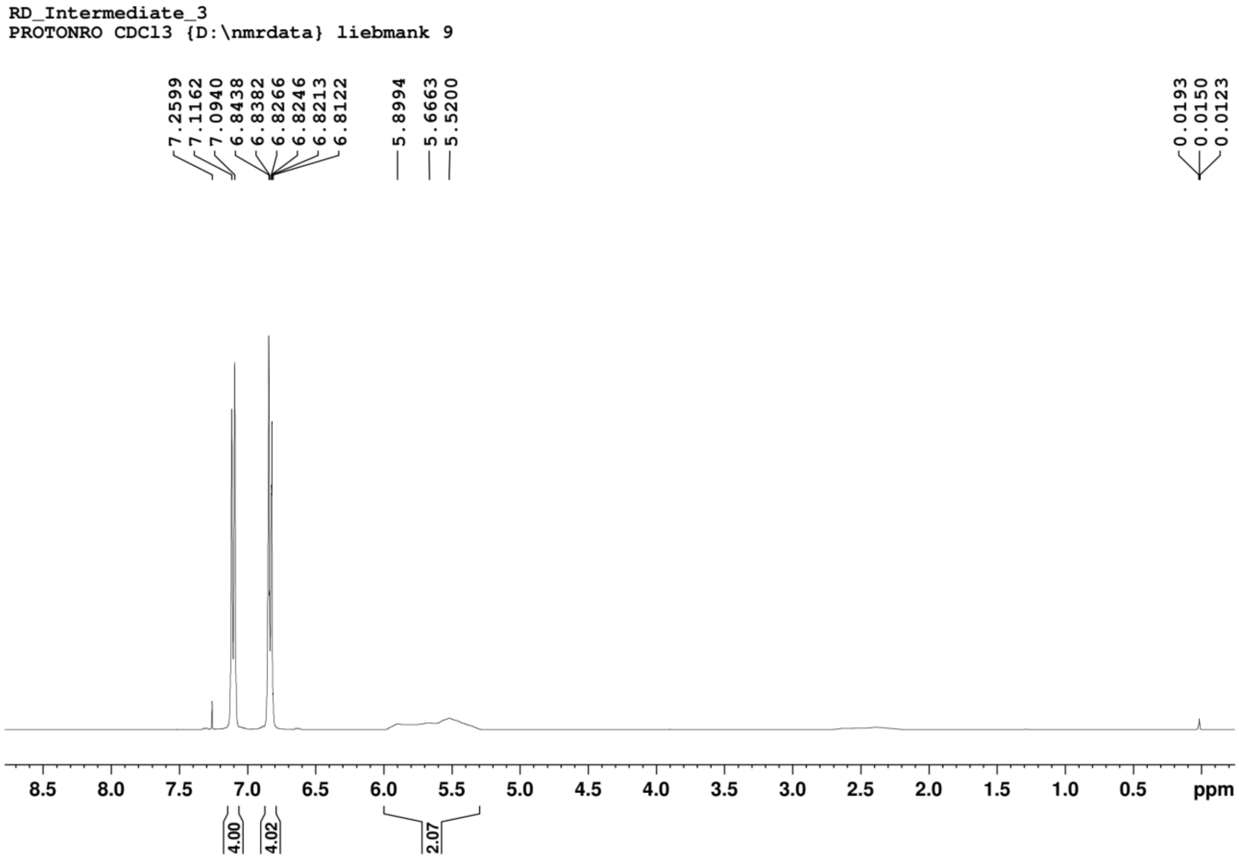

Supplementary Figure 5. ^{1}H NMR spectrum of intermediate **3** ($CDCl_3$, 400 MHz).

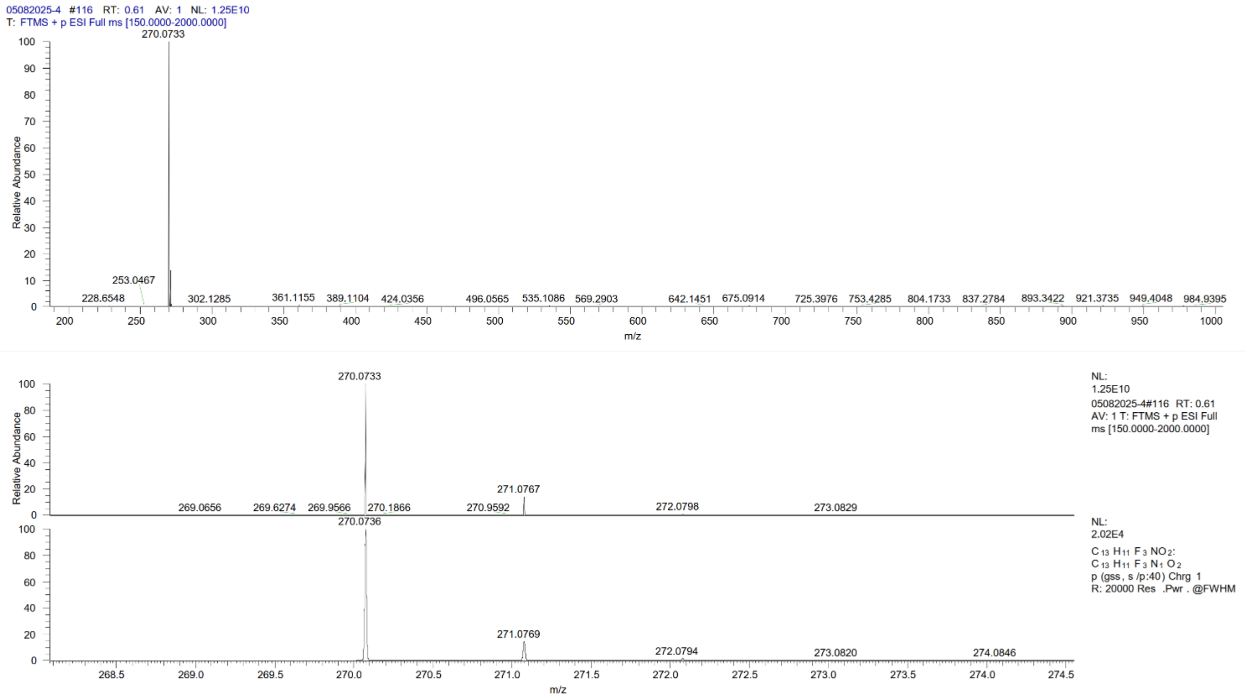

Supplementary Figure 6. HRMS (ESI) of intermediate **3**; *m/z*: $[M + H]^+$ Calcd for $C_{13}H_{11}F_3NO_2$ 270.0736; Found 270.0733.

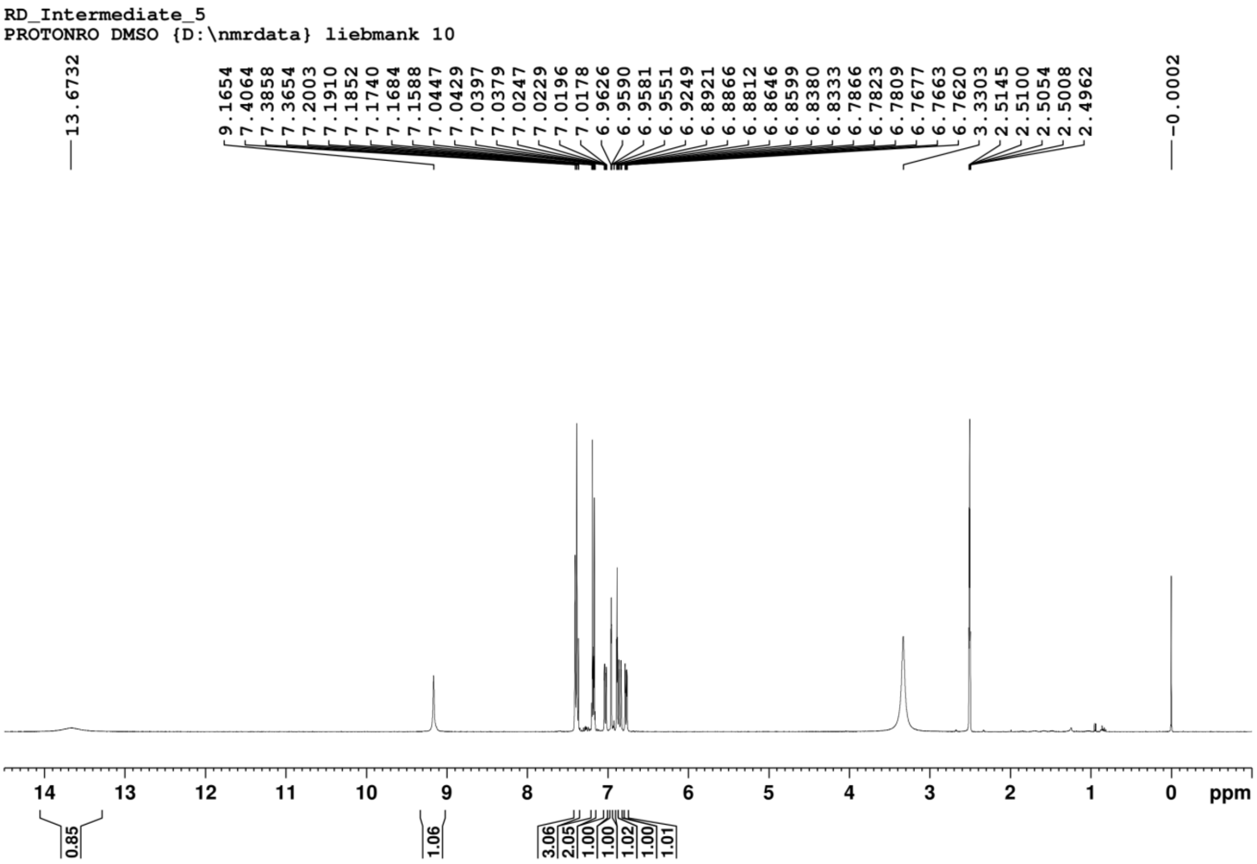

Supplementary Figure 7. ^{1}H NMR spectrum of intermediate **5** (DMSO-d_6, 400 MHz).

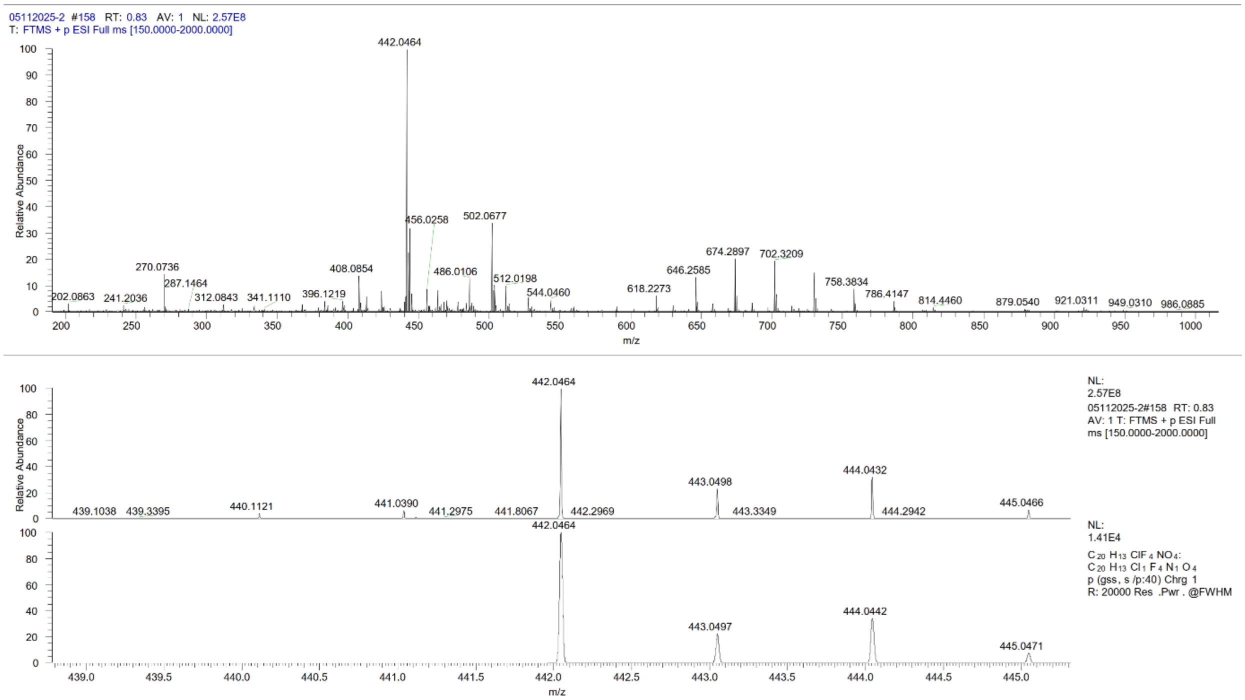

Supplementary Figure 8. HRMS (ESI) of intermediate **5**; *m/z*: $[M + H]^+$ Calcd for $C_{20}H_{13}ClF_4NO_4$ 442.0464; Found 442.0464.

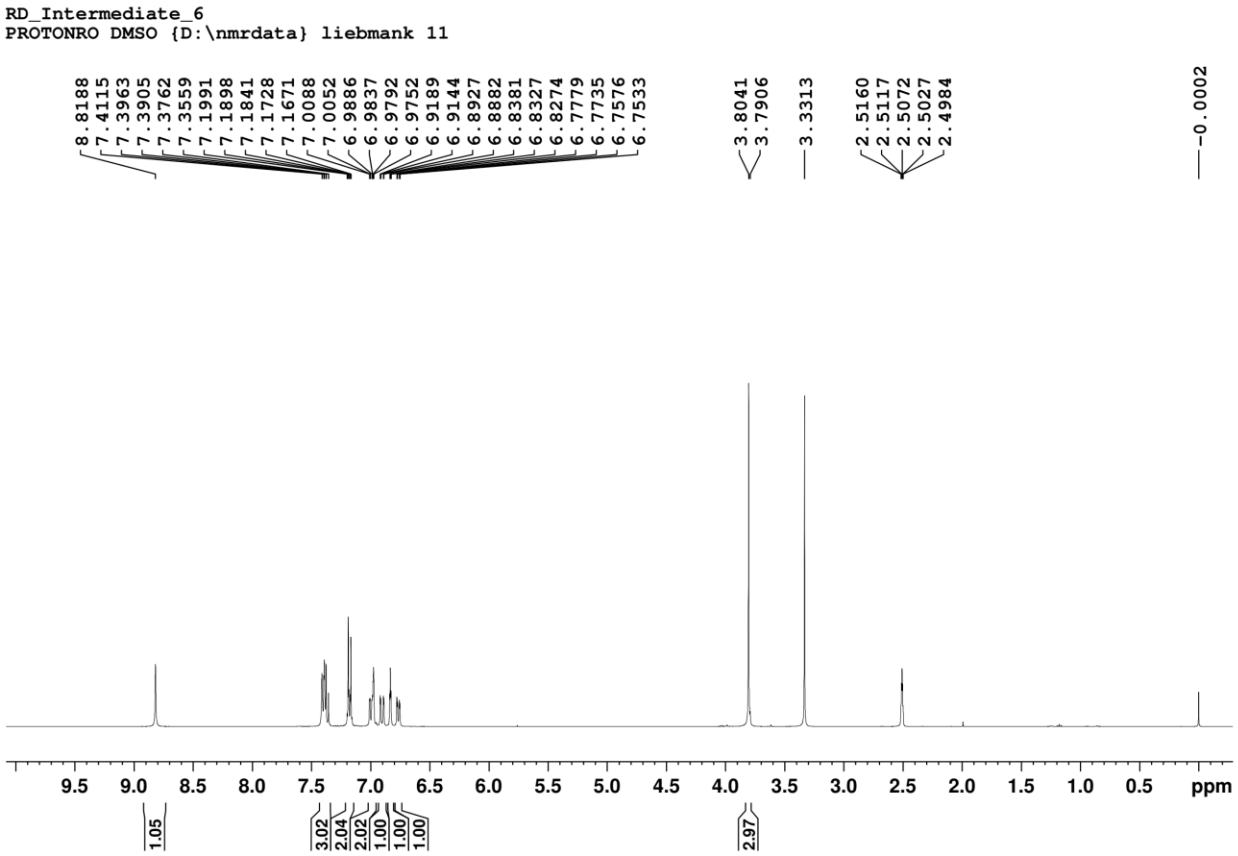

Supplementary Figure 9. ^{1}H NMR spectrum of intermediate **6** (DMSO-d_6, 400 MHz).

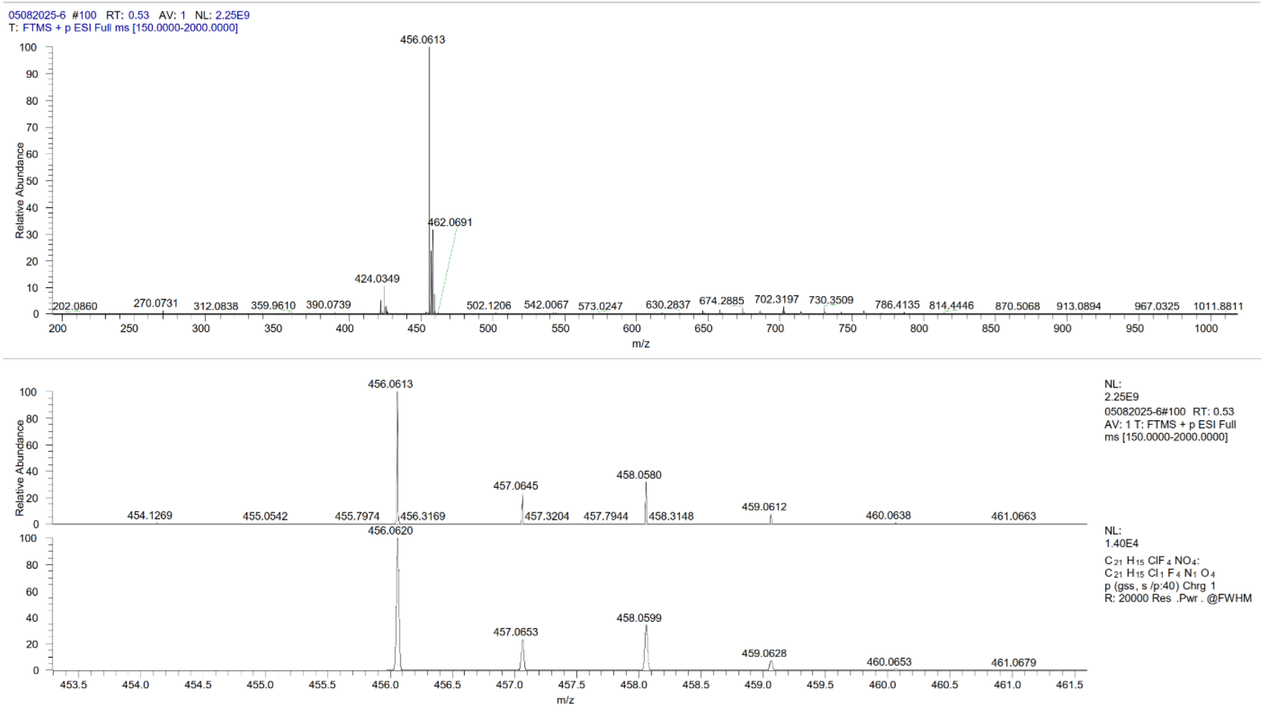

Supplementary Figure 10. HRMS (ESI) of intermediate **6**; *m/z*: $[M + H]^+$ Calcd for $C_{21}H_{15}ClF_4NO_4$ 456.0620; Found 456.0613

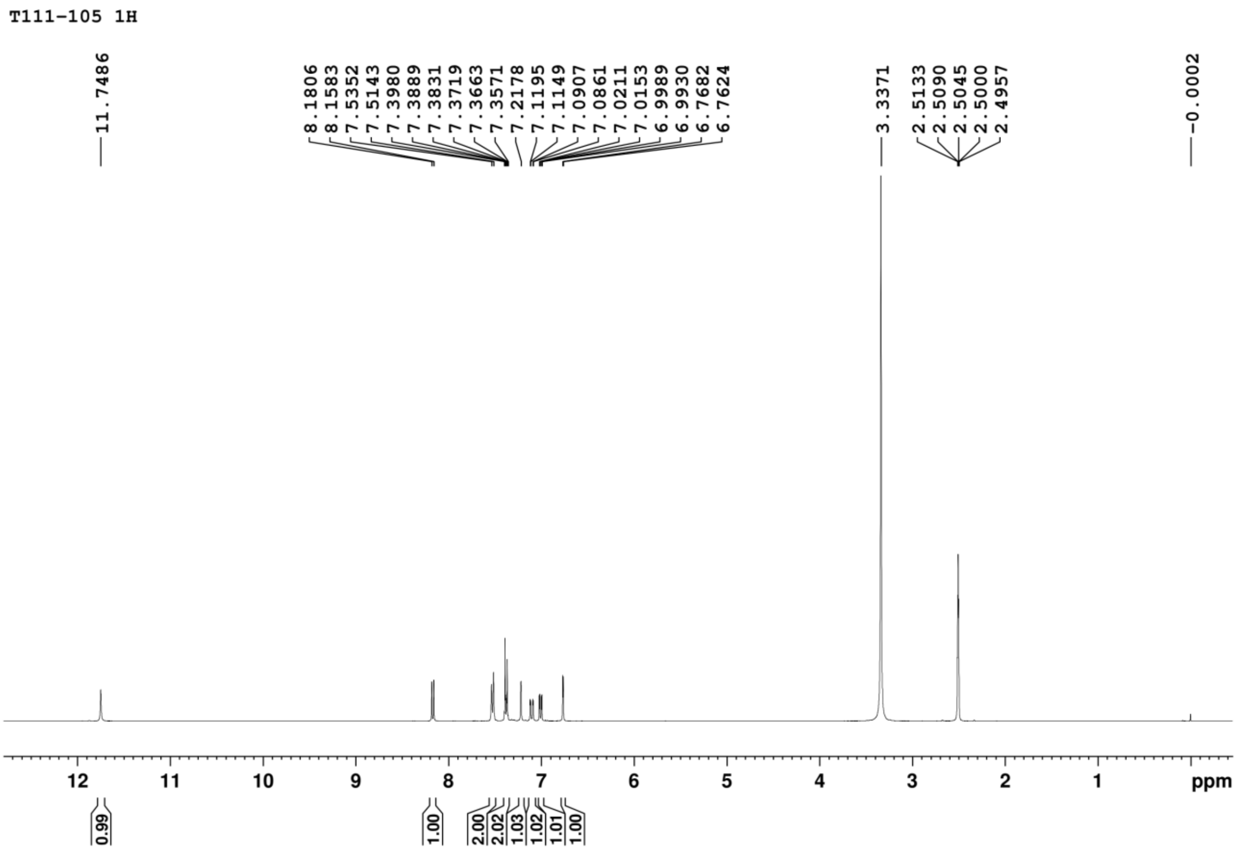

Supplementary Figure 11. ^{1}H NMR spectrum of T111 (DMSO-d_6, 400 MHz).

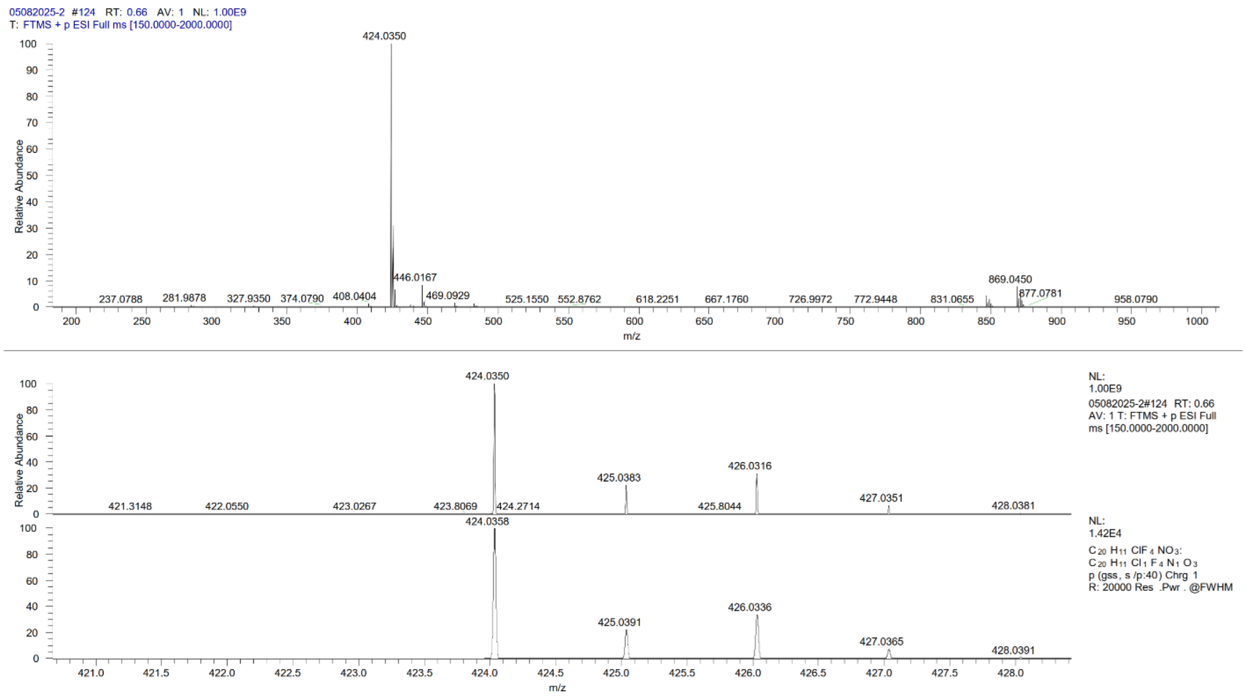

Supplementary Figure 12. HRMS (ESI) of T111; *m/z*: $[M + H]^+$ Calcd for $C_{20}H_{11}ClF_4NO_3$ 424.0358; Found 424.0350.

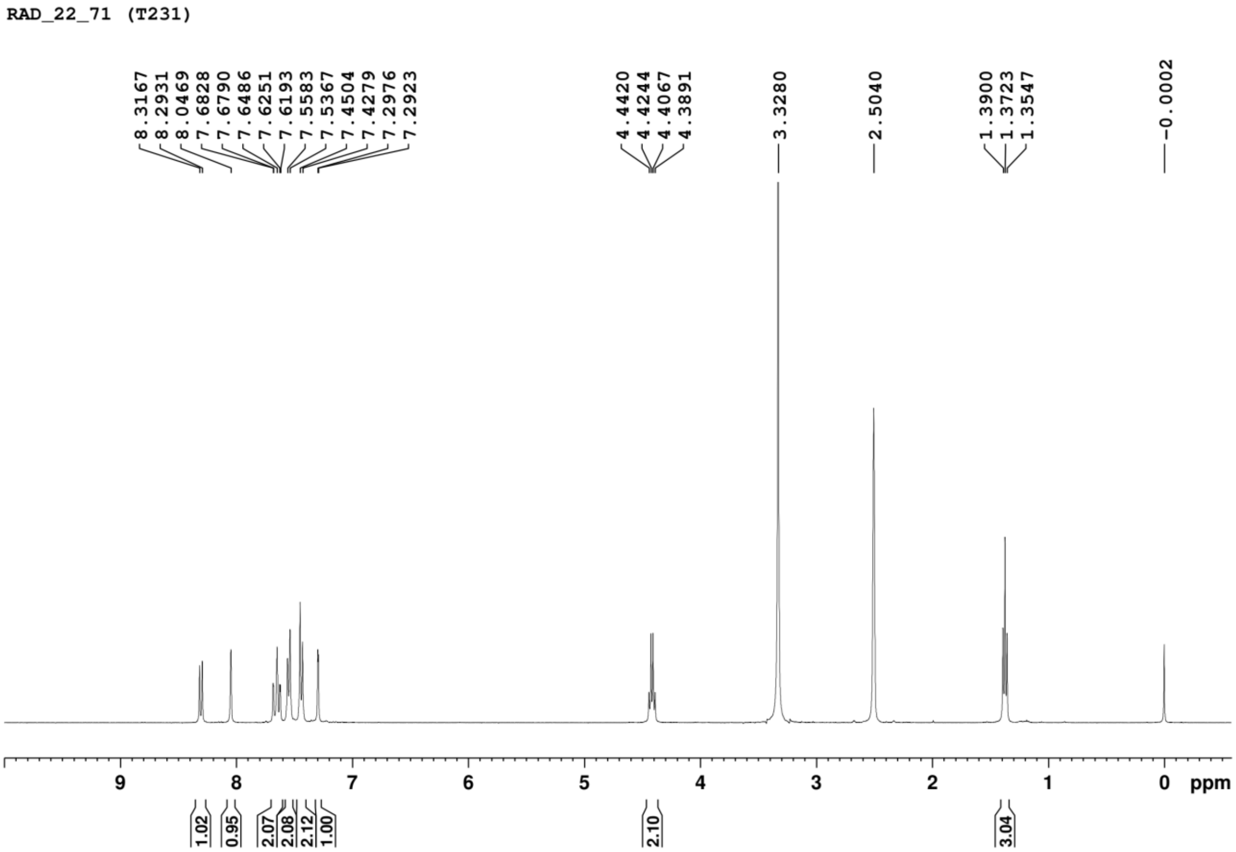

Supplementary Figure 13. ^{1}H NMR spectrum of T231 (DMSO-d_6, 400 MHz).

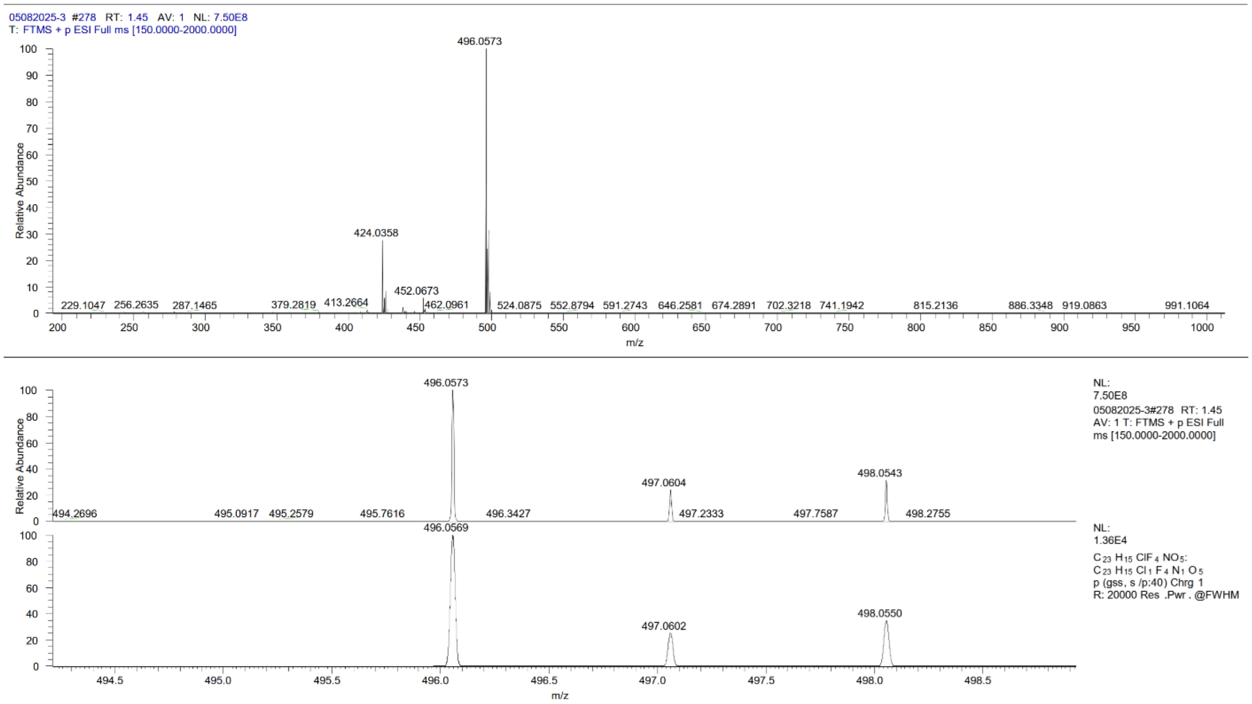

Supplementary Figure 14. HRMS (ESI) of T231; *m/z*: $[M + H]^+$ Calcd for $C_{23}H_{15}ClF_4NO_5$ 496.0569; Found 496.0573.
