# Supplementary material for: Potent acridone antimalarial against all three life stages of Plasmodium
Source: Nat Commun. 2026 Apr 14;17:4230. doi: 10.1038/s41467-026-71708-1 (PMC13168289; doi:10.1038/s41467-026-71708-1)
Supplement: Supplementary file 3 — Reporting Summary [file 41467_2026_71708_MOESM3_ESM.pdf]

Corresponding author(s): Jane X. Kelly and Papireddy Kancharla

Last updated by author(s): Mar 23, 2026

# Reporting Summary

Nature Portfolio wishes to improve the reproducibility of the work that we publish. This form provides structure for consistency and transparency in reporting. For further information on Nature Portfolio policies, see our Editorial Policies and the Editorial Policy Checklist.

## Statistics

For all statistical analyses, confirm that the following items are present in the figure legend, table legend, main text, or Methods section.

| n/a | Confirmed | |
|---|---|---|
| ☐ | ☒ | The exact sample size (*n*) for each experimental group/condition, given as a discrete number and unit of measurement |
| ☐ | ☒ | A statement on whether measurements were taken from distinct samples or whether the same sample was measured repeatedly |
| ☐ | ☒ | The statistical test(s) used AND whether they are one- or two-sided<br>*Only common tests should be described solely by name; describe more complex techniques in the Methods section.* |
| ☐ | ☒ | A description of all covariates tested |
| ☐ | ☒ | A description of any assumptions or corrections, such as tests of normality and adjustment for multiple comparisons |
| ☐ | ☒ | A full description of the statistical parameters including central tendency (e.g. means) or other basic estimates (e.g. regression coefficient) AND variation (e.g. standard deviation) or associated estimates of uncertainty (e.g. confidence intervals) |
| ☐ | ☒ | For null hypothesis testing, the test statistic (e.g. *F*, *t*, *r*) with confidence intervals, effect sizes, degrees of freedom and *P* value noted<br>*Give P values as exact values whenever suitable.* |
| ☒ | ☐ | For Bayesian analysis, information on the choice of priors and Markov chain Monte Carlo settings |
| ☒ | ☐ | For hierarchical and complex designs, identification of the appropriate level for tests and full reporting of outcomes |
| ☐ | ☒ | Estimates of effect sizes (e.g. Cohen's *d*, Pearson's *r*), indicating how they were calculated |

*Our web collection on statistics for biologists contains articles on many of the points above.*

## Software and code

Policy information about availability of computer code

| | |
|---|---|
| Data collection | • Imaging and Plate Reading: Images for liver-stage parasite quantification were acquired using Harmony 4.9 (PerkinElmer) on an Operetta CLS high-content imaging system. Bioluminescence for in vivo imaging was collected using the IVIS system.<br>• Flow Cytometry: Parasitemia and eryptosis data were collected using FACSCalibur instrumentation.<br>• qPCR: Cycle threshold (Ct) data for the GRRA50 assays were calculated using AB1 SDA 2.4.1 software.<br>• Spectroscopy: Proton NMR spectra were recorded on a Bruker AMX-400 spectrometer at 400 MHz, and high-resolution mass spectra (HRMS) (electrospray ionization (ESI)) were recorded on a vanquish UHPLC/HPLC system coupled with a high resolution (35,000) Q Exactive Orbitrap mass spectrometer. |
| Data analysis | • Genomic Analysis: Sequence data were aligned to the P. falciparum Dd2 genome using Bowtie 2 (v2.4.2). Sorting was performed with samtools (v1.11), duplicates were marked with picard (v2.25.0), and variants were called using freebayes (v1.3.5). Variant annotation was performed with SnpEff (v5.0). Sanger sequences were analyzed using 4Peaks and Clustal Omega.<br>• Molecular Modeling & Docking: Automated docking simulations and Qo binding site modeling were conducted using AutoDock 4.0.<br>• Pharmacokinetics & Toxicokinetics: Noncompartmental PK and TK parameters were analyzed using Phoenix WinNonlin (versions 6.4 and 8.3).<br>• Statistical Analysis & Graphing: All non-linear regressions, IC50/ED50 calculations, and dose-response curves were generated using GraphPad Prism (versions 8.0, 9.0, 10, and 10.5.0). Microsoft Excel (version 16.66.1) was used for data organization, FIC index calculations, and the generation of isobolograms.<br>• Chemical and Structural Tools: NMR data were processed and analyzed using TopSpin (version 4.4.1). HRMS data were processed and analyzed using Thermo Xcalibur Qual Browser (version 4.1.50). ChemDraw (version 23.1.1) was used to generate all chemical structures and synthetic schemes. |

• No custom code was used, and we have provided the raw replicates and curves as Source Data to ensure full transparency and reproducibility.

For manuscripts utilizing custom algorithms or software that are central to the research but not yet described in published literature, software must be made available to editors and reviewers. We strongly encourage code deposition in a community repository (e.g. GitHub). See the Nature Portfolio guidelines for submitting code & software for further information.

## Data

Policy information about availability of data

All manuscripts must include a data availability statement. This statement should provide the following information, where applicable:

- Accession codes, unique identifiers, or web links for publicly available datasets
- A description of any restrictions on data availability
- For clinical datasets or third party data, please ensure that the statement adheres to our policy

Source data for all figures and tables are provided with this paper. The raw and processed data generated in this study have been deposited in the Figshare database under accession code 31825489 (https://doi.org/10.6084/m9.figshare.31825489). The Whole Genome Sequencing (WGS) data generated in this study have been deposited in the NCBI BioProject database under accession code PRJNA1417775 (https://www.ncbi.nlm.nih.gov/bioproject/PRJNA1417775), which contains seven individual SRA records (SRX32021460–SRX32021466) corresponding to the P. falciparum Dd2 parental and derived mutant lines analyzed in this study. No data in this study are under restricted access or protected by data privacy laws.

## Research involving human participants, their data, or biological material

Policy information about studies with human participants or human data. See also policy information about sex, gender (identity/presentation), and sexual orientation and race, ethnicity and racism.

**Reporting on sex and gender**

• In vitro GRRA Susceptibility Testing of P. falciparum Clinical Isolates (Rwanda)
o Findings from these isolates apply to both sexes.
o Participant gender was determined based on self-reporting during the original clinical recruitment in 2019.
o The study population consisted of 51.3% female and 48.7% male participants.
o Sex- and gender-based analyses were not performed specifically for these in vitro susceptibility assays as the primary objective was the characterization of parasite-encoded resistance markers (K13 mutations).
• Ex vivo Susceptibility Testing of African P. falciparum Isolates (Burkina Faso and Uganda)
o The study utilized clinical isolates derived from both male and female patients. In the Burkina Faso and Uganda longitudinal studies, both male and female patients were recruited based on clinical presentation of uncomplicated malaria.
o Sex and gender were determined based on self-reporting by the participants (or their legal guardians) at the time of initial clinical enrollment at the respective health centers.
o As the focus was on the biological characteristics of the P. falciparum parasites rather than host physiological responses, sex- and gender-based analyses were not performed. The findings regarding the activity of studied compounds are expected to apply equally across all genders.
• In Vitro Blood Stage Susceptibility Testing (Lampire Biological Laboratories)
o This study component utilized de-identified human red blood cells (RBCs) sourced from Lampire Biological Laboratories (Pipersville, PA, USA).
o While the biological materials were derived from a donor pool including both sexes, sex- and gender-based analyses were not performed. The RBCs serve as a standardized host cell for in vitro parasite cultivation and drug-susceptibility assays, where the sex of the original donor is not a biological variable that impacts the metabolic or growth-inhibition outcomes of the P. falciparum assays.
• Standard Membrane Feeding Assays (University of Melbourne)
o This component utilized de-identified human blood products (red blood cells and serum) sourced from Australian Red Cross Lifeblood donors of all genders.
o Sex- and gender-based analyses were not performed, as the biological materials were used as a standardized substrate for in vitro parasite cultivation and mosquito feeding. Host sex is not a biological variable that impacts the metabolic or developmental outcomes of the SMFA drug-susceptibility assays.
• Eryptosis Assays — Volunteer-Derived Human Erythrocytes (WRAIR)
o This study component utilized de-identified human erythrocytes (normal and G6PD-deficient RBCs) derived from adult volunteers.
o Both male and female donors were included in the specimen collection.
o Sex- and gender-based analyses were not performed, as the research focused on the biochemical and cellular response of the erythrocytes (eryptosis) to compound exposure. The findings regarding the activity of studied compounds are expected to apply across all genders.

**Reporting on race, ethnicity, or other socially relevant groupings**

• In vitro GRRA Susceptibility Testing of P. falciparum Clinical Isolates (Rwanda)
o The study utilized indigenous clinical isolates from patients in the Huye District, Southern Province, Rwanda.
o Regional origin and African ancestry of the isolates (SP045 and SP060) were confirmed using 23-SNP barcoding and haplotype analysis (Haplotype 22).
• Ex vivo Susceptibility Testing of African P. falciparum Isolates (Burkina Faso and Uganda)
o The research utilized indigenous P. falciparum clinical isolates collected from patients in Bobo-Dioulasso (Burkina Faso), and the Tororo, Busia, Mbale, and Agago districts (Uganda).
o Participants were grouped by geographical origin to monitor the regional emergence and spread of antimalarial resistance markers (such as pfk13 mutations) in East and West Africa.
o The African ancestry and regional genetic signatures of the isolates were confirmed through molecular inversion probe (MIP) capture and deep sequencing of 80 P. falciparum genes to ensure the results are representative of the local parasite

populations in these malaria-endemic regions.
• In Vitro Blood Stage Susceptibility Testing (Lampire Biological Laboratories)
o Reporting on race, ethnicity, or other socially relevant groupings is not applicable to this study component. The biological materials (RBCs) were de-identified commercial products sourced from a general donor population for laboratory research use.
• Standard Membrane Feeding Assays (University of Melbourne)
o Reporting on race, ethnicity, or other socially relevant groupings is not applicable to this study component. The biological materials (blood and serum) were de-identified products sourced from the general donor population of a national blood service.
• Eryptosis Assays — Volunteer-Derived Human Erythrocytes (WRAIR)
o The study utilized erythrocytes from a diverse pool of adult volunteers.
o Specific reporting on race or ethnicity was not conducted; however, the inclusion of G6PD-deficient samples was essential to evaluate the safety profile of the studied compounds in a clinically relevant biological context.

Population characteristics

• In vitro GRRA Susceptibility Testing of P. falciparum Clinical Isolates (Rwanda)
o Isolates were derived from symptomatic patients with microscopically confirmed uncomplicated P. falciparum malaria.
o The median age of the participant cohort was 18 years, with an age range of 2 to 69 years.
o Genotypic information: The selected isolates (SP045 and SP060) harbor validated or candidate Kelch13 propeller domain mutations, specifically V555A, or R561H, respectively.
• Ex vivo Susceptibility Testing of African P. falciparum Isolates (Burkina Faso and Uganda)
o Symptomatic patients with uncomplicated malaria and P. falciparum mono-infection.
o Burkina Faso Cohort: Patients seeking treatment at the Colsam Urban Health Centre in Bobo-Dioulasso during the 2021 and 2022 malaria transmission seasons.
o Uganda Cohort: Patients seeking treatment at government health facilities in the Tororo, Busia, Mbale, and Agago districts (2010–2024).
o Parasitemia: Inclusion for ex vivo testing generally required a parasitemia range of 2,000–200,000 parasites/µL.
• In Vitro Blood Stage Susceptibility Testing (Lampire Biological Laboratories)
o This research component involved the use of de-identified human RBCs for the in vitro cultivation and testing of P. falciparum.
o Because no human research participants were directly recruited by the investigators for this component, specific demographic characteristics such as age, clinical diagnosis, or participant genotype are not applicable.
• Standard Membrane Feeding Assays (University of Melbourne)
o This research component involved the use of de-identified human blood products for the in vitro cultivation of P. falciparum and subsequent mosquito feeding.
o Because no human research participants were directly recruited by the investigators for this assay, specific demographic characteristics such as age, clinical diagnosis, or participant genotype are not applicable.
• Eryptosis Assays — Volunteer-Derived Human Erythrocytes (WRAIR)
o Donor Status: Adult volunteers in stable health.
o Genotypic Profile: The study specifically utilized de-identified samples of both normal (wild-type) erythrocytes and G6PD-deficient erythrocytes to assess potential hemolytic risk and oxidative stress (eryptosis) induced by acridone lead candidates.

Recruitment

• In vitro GRRA Susceptibility Testing of P. falciparum Clinical Isolates (Rwanda)
o Participants were prospectively recruited between September and December 2019 upon seeking treatment at the Sovu Health Centre and Kabutare District Hospital in Huye, Rwanda.
o Potential self-selection bias was minimized by enrolling consecutive eligible patients seeking treatment for malaria symptoms during the defined study period.
o Enrollment was predicated on positive rapid diagnostic test (RDT) results and measured axillary temperature > 37.5 °C.
• Ex vivo Susceptibility Testing of African P. falciparum Isolates (Burkina Faso and Uganda)
o Participants were recruited prospectively upon seeking treatment at the Colsam Urban Health Centre (Bobo-Dioulasso, Burkina Faso) or government health facilities in the Tororo, Busia, Mbale, and Agago districts (Uganda). Inclusion was based on presentation with signs of malaria and microscopic confirmation of P. falciparum. Selection bias was minimized by enrolling consecutive eligible patients who met the parasitemia requirements for ex vivo testing (typically 2,000–200,000 parasites/µL).
• In Vitro Blood Stage Susceptibility Testing (Lampire Biological Laboratories)
o De-identified human RBCs were purchased from Lampire Biological Laboratories (Pipersville, PA).
o Materials were collected by the supplier from voluntary donors in accordance with the supplier's internal regulatory and ethical protocols for biological material collection for research purposes.
o No specific recruitment of human participants was performed by the study investigators.
• Standard Membrane Feeding Assays (University of Melbourne)
o De-identified human blood and serum were provided by Australian Red Cross Lifeblood.
o Materials were collected by Lifeblood from voluntary donors in accordance with standard national regulatory and ethical protocols for blood donation.
o The study investigators did not perform any specific recruitment of human participants for this laboratory-based component.
• Eryptosis Assays — Volunteer-Derived Human Erythrocytes (WRAIR)
o De-identified specimens were collected from adult volunteers as part of a dedicated collection protocol at the Walter Reed Army Institute of Research (WRAIR).
o Enrollment was voluntary, and participants were recruited through institutional internal advertisements for blood donation for research purposes.
o All donors met standard institutional health and eligibility criteria for blood collection.

Ethics oversight

• In vitro GRRA Susceptibility Testing of P. falciparum Clinical Isolates (Rwanda)
o The original collection study and the use of these biological materials were approved by the Rwanda National Ethics Committee, Ref no. 416/RNEC/2017 and 686/RNEC/2019.
o Written informed consent was obtained from all adult participants or from caregivers for child participants.
o Written assent was obtained from all minor participants between the ages of 7 and 18 years.
• Ex vivo Susceptibility Testing of African P. falciparum Isolates (Burkina Faso and Uganda)
o Ethical approvals were granted by the Institutional Ethics Committee of the Institut de Recherche en Sciences de la Santé (IRSS) (Bobo-Dioulasso, Burkina Faso), the Makerere University School of Biomedical Sciences Research Ethics Committee (Kampala, Uganda), and the Uganda National Council for Science and Technology. Written informed consent was obtained from all adult participants or from the parents or legal guardians of child participants. Written assent was also obtained from minor participants (aged 8–17 years) in accordance with national guidelines.
• In Vitro Blood Stage Susceptibility Testing (Lampire Biological Laboratories)
o The use of commercially sourced, de-identified human RBCs for in vitro laboratory research is exempt from formal Institutional Review Board (IRB) review under federal regulation 45 CFR 46, as the investigators cannot identify the donors.
o All laboratory work was conducted in compliance with the safety and ethical guidelines for handling human-derived biological materials at the performing institution.
• Standard Membrane Feeding Assays (University of Melbourne)
o Approving Body: University of Melbourne Human Research Ethics Committee (HREC).
o Project ID: 22013.
o The use of these blood products was conducted in accordance with the ethical guidelines of the University of Melbourne and specific deeds of agreement with Australian Red Cross Lifeblood.
• Eryptosis Assays — Volunteer-Derived Human Erythrocytes (WRAIR)
o Approving Body: Ethics Committee of the Walter Reed Army Institute of Research (WRAIR).
o Protocol Information: Specimens were collected under protocol #2567.04, titled "Blood Collection for G6PD Antimalarial Assays" (version 1.0, approved December 21, 2018).
o Consent: Written informed consent was obtained from all participants prior to blood collection in accordance with institutional and federal regulations (45 CFR 46). All samples were de-identified prior to laboratory analysis.
o Protocol Availability: In compliance with U.S. Department of Defense and institutional security policies, specific internal protocol numbers are maintained by the USAMD-AFRIMS IACUC Office and are available for independent verification by the Editorial Office upon request.

Note that full information on the approval of the study protocol must also be provided in the manuscript.

# Field-specific reporting

Please select the one below that is the best fit for your research. If you are not sure, read the appropriate sections before making your selection.

☒ Life sciences ☐ Behavioural & social sciences ☐ Ecological, evolutionary & environmental sciences

For a reference copy of the document with all sections, see nature.com/documents/nr-reporting-summary-flat.pdf

# Life sciences study design

All studies must disclose on these points even when the disclosure is negative.

Sample size

• In vivo blood-stage efficacy studies (VAPORHCS)
o Sample sizes for in vivo blood-stage efficacy studies were determined based on established protocols for the 4-day suppression and single-dose cure models.
o Groups of n=4 mice (CF1 strain) were utilized per dose group. This sample size was determined to provide sufficient statistical power (0.80) to detect a 90% reduction in parasitemia (ED90) relative to drug-free controls, based on historical effect sizes in the P. yoelii (Kenya strain) model.
• In vivo liver-stage efficacy and PK studies (WRAIR)
o Sample sizes for in vivo efficacy and pharmacokinetic (PK) studies were determined by a priori power analysis using G*Power software (version 3.1.9.7).
o Determination was based on effect sizes from previous experiments to ensure a power of 0.80 and alpha of 0.05.
o PK studies used n=3 mice per sex (total n=6 per dose group).
o Efficacy studies utilized n=5 mice per group for liver-stage efficacy studies.
• In vivo toxicology and toxicokinetic (TK) studies (SRI International)
o Sample sizes were determined based on regulatory standards and generally accepted drug development practices for Maximum Tolerated Dose (MTD) and range-finding repeat-dose toxicology studies.
o The MTD study utilized n=2 male and n=2 female Sprague Dawley rats per dose group (30, 100, 200, or 400 mg/kg).
o The 7-day repeat-dose study utilized n=8 male and n=8 female Sprague Dawley rats per group (25, 100, or 400 mg/kg, and vehicle control) to provide sufficient power for clinical pathology, organ weight analysis, and histopathology.
Detailed statements regarding sample size determination and the statistical parameters used have been included in the "Statistical analysis and sample size determination" subsection of the Methods.

Data exclusions

• In vivo blood-stage efficacy studies (VAPORHCS)
o No data were excluded from the analyses. All animals infected and treated within the 4-day suppression and single-dose cure cohorts were included in the final parasitemia calculations and cure rate determinations reported in the manuscript.

• In vivo liver-stage efficacy and PK studies (WRAIR)
o No data were excluded from the analyses.
o All experimental results, including technical replicates and all animals utilized in the in vivo studies, are reported in the manuscript and source data files.
• In vivo toxicology and toxicokinetic (TK) studies (SRI International)
o No data were excluded from the analyses. All animals enrolled in the MTD and 7-day repeat-dose studies were included in the clinical observations, pathology, and toxicokinetic data sets reported in the manuscript.
All experimental results, including technical replicates and all animals utilized in the in vivo studies, are reported in the manuscript and source data files.

Replication

• In vivo blood-stage efficacy studies (VAPORHCS)
o The in vivo efficacy experiments were performed in independent runs to confirm the activity of T111 and the T111/TQ combinations. All attempts at replication were successful, with consistent ED50 and ED90 values observed across experimental replicates. The specific number of replicates is indicated in the corresponding figure legends and tables.
• In vivo liver-stage efficacy and PK studies (WRAIR)
o All attempts at replication were successful.
o In vivo efficacy experiments were repeated independently to confirm lead compound activity.
o The specific number of biological and technical replicates for each experiment is provided in the corresponding figure legends.
• In vivo toxicology and toxicokinetic (TK) studies (SRI International)
o Measurements of drug plasma levels, clinical pathology and histopathology were conducted on multiple animals per group (including both males and females in all experimental groups) to confirm the consistency of the safety observations.
The specific number of biological and technical replicates for each experiment is provided in the corresponding figure legends.

Randomization

• In vivo blood-stage efficacy studies (VAPORHCS)
o Four- to five-week-old female and male CF1 mice were randomly assigned to treatment and control groups using a simple randomization procedure (computer-generated random numbers). This ensured a balanced distribution of baseline body weights (mean ~30 g) and starting parasitemia levels across all experimental cohorts.
• In vivo liver-stage efficacy and PK studies (WRAIR)
o Mice were randomly assigned to treatment and control groups using a simple randomization method (random number generation) after confirming baseline health and weight.
• In vivo toxicology and toxicokinetic (TK) studies (SRI International)
o Male and female Sprague Dawley rats were randomly assigned to treatment and control groups using a simple randomization procedure. This process ensured a balanced distribution of baseline body weights across all study cohorts prior to the commencement of dosing.

Blinding

• In vivo blood-stage efficacy studies (VAPORHCS)
o Investigators were not blinded to the group assignments during the oral gavage administration of the compounds. However, the primary endpoint (parasitemia levels) was determined through microscopic examination of Giemsa-stained thin blood films. To minimize bias, slides were coded and read by experienced technicians who were blinded to the specific treatment groups during the counting process.
• In vivo liver-stage efficacy and PK studies (WRAIR)
o Blinding was not implemented during drug administration.
o To minimize investigator bias, parasitemia quantification was performed using automated bioluminescence imaging or flow cytometry.
• In vivo toxicology and toxicokinetic (TK) studies (SRI International)
o Investigators were not blinded to the dose group assignments during drug administration, clinical observations, or any other analyses conducted in support of the toxicology study. However, clinical pathology (hematology and chemistry) and microscopic examinations of tissues for histopathology were performed using standardized, objective criteria. Plasma drug levels were determined using a bioanalytical method that employed objective measurements relative to a standard curve of T111. Blinding was not implemented during drug administration.

# Reporting for specific materials, systems and methods

We require information from authors about some types of materials, experimental systems and methods used in many studies. Here, indicate whether each material, system or method listed is relevant to your study. If you are not sure if a list item applies to your research, read the appropriate section before selecting a response.

## Materials & experimental systems

| n/a | Involved in the study |
|---|---|
| ☒ | ☐ Antibodies |
| ☐ | ☒ Eukaryotic cell lines |
| ☒ | ☐ Palaeontology and archaeology |
| ☐ | ☒ Animals and other organisms |
| ☒ | ☐ Clinical data |
| ☒ | ☐ Dual use research of concern |
| ☒ | ☐ Plants |

## Methods

| n/a | Involved in the study |
|---|---|
| ☒ | ☐ ChIP-seq |
| ☒ | ☐ Flow cytometry |
| ☒ | ☐ MRI-based neuroimaging |

# Eukaryotic cell lines

Policy information about cell lines and Sex and Gender in Research

| | |
|---|---|
| Cell line source(s) | • HepG2 cells (human hepatocellular carcinoma) were purchased from ATCC.<br>• All strains of Plasmodium falciparum and Plasmodium yoelii were obtained from BEI Resources.<br>• Luciferase and GFP-expressing Plasmodium berghei ANKA (MRA-868) were obtained from the Malaria Research and Reference Reagent Resource Center (MR4), currently residing with BEI Resources.<br>• Plasmodium cynomolgi bastianellii (B strain) infected Anopheles dirus mosquitoes are maintained at AFRIMS and shipped to WRAIR. |
| Authentication | • HepG2 and parasite strain identities were authenticated by the suppliers (ATCC and BEI) using morphology, karyotyping, and PCR-based approaches.<br>• For all parasite lines, in vitro susceptibility tests were performed to confirm that the drug resistance profile (IC50 values) matched the expected phenotype of the specific strain. |
| Mycoplasma contamination | All cell lines utilized in this study were tested for mycoplasma contamination and confirmed to be negative. |
| Commonly misidentified lines<br>(See ICLAC register) | No commonly misidentified cell lines (as defined by the ICLAC register) were utilized in this study. |

# Animals and other research organisms

Policy information about studies involving animals; ARRIVE guidelines recommended for reporting animal research, and Sex and Gender in Research

| | |
|---|---|
| Laboratory animals | • In vivo blood-stage efficacy studies (VAPORHCS)<br>o Species/Strain: Mice (Mus musculus), outbred CF1 strain (~30 g) from from Charles River Laboratories. .<br>o Age/Sex: 4- to 5-week-old female and male mice.<br>o Husbandry: Animals were housed in Thoren Maxi-Miser microisolator cages (78 sq. in. floor space) with a maximum density of five females or four males per cage. Cages were maintained on a 12-h light/dark cycle and provided with autoclaved nesting material (nestlets) for environmental enrichment. All mice had ad libitum access to food and water.<br>• In vivo liver-stage efficacy studies (WRAIR)<br>o Species/Strain: Mice (Mus musculus), inbred Albino C57BL/6 strain (18–22 g) from Jackson Laboratories.<br>o Age/Sex: 4- to 6-week-old female mice.<br>o Husbandry: Animals were maintained in an AAALAC International-accredited facility. All animals were quarantined and acclimatized for 7 days prior to study initiation and were housed in a 12:12 hour light/dark cycle with food and water provided ad libitum. The housing environment was maintained at a temperature of 18–26 °C (64–79 °F) with relative humidity between 34% and 68%. The experiments reported herein were conducted in compliance with the Animal Welfare Act and per the principles set forth in the "Guide for Care and Use of Laboratory Animals," Institute of Laboratory Animals Resources, National Research Council, National Academy Press, 1996.<br>• In vivo PK studies (WRAIR)<br>o Species/Strain: Mice (Mus musculus), outbred ICR-CD1 mice (23–35 g) from Charles River Laboratories.<br>o Age/Sex: Adult mice (4–5 weeks of age); both males and females were used for PK, and females were used for liver-stage efficacy.<br>o Husbandry: Animals were maintained in an AAALAC International-accredited facility. All animals were quarantined and acclimatized for 7 days prior to study initiation and were housed in a 12:12 hour light/dark cycle with food and water provided ad libitum. The housing environment was maintained at a temperature of 18–26 °C (64–79 °F) with relative humidity between 34% and 68%. The experiments reported herein were conducted in compliance with the Animal Welfare Act and per the principles set forth in the "Guide for Care and Use of Laboratory Animals," Institute of Laboratory Animals Resources, National Research Council, National Academy Press, 1996.<br>• In vivo toxicology and toxicokinetic (TK) studies (SRI International)<br>o Species/Strain: Rats (Rattus norvegicus), outbred Sprague Dawley strain from Charles River Laboratories.<br>o Age/Sex: Adult males (300–400 g) and females (225–275 g).<br>o Husbandry: Rats were housed in microisolator cages with hardwood chip bedding (Sani-Chips) and maintained on a 12-h light/dark cycle (20–26°C; 30–70% relative humidity). Animals were group-housed with ad libitum access to a certified global 18% protein rodent diet and purified water. |
| Wild animals | N/A |
| Reporting on sex | • Study Design and Analysis: Sex was considered a key biological variable in the design of efficacy, pharmacokinetic (PK), and toxicokinetic (TK) studies.<br>• Pharmacokinetics (PK) and Toxicokinetics (TK): Equal cohorts of male and female animals were utilized to assess potential sex-based differences in drug exposure and metabolism. Specifically, Sprague Dawley rats were evaluated in both sexes for TK parameters (Supplementary Table 3), and both male and female ICR-CD1 mice were used for PK evaluations (Table 3).<br>• Blood-Stage Efficacy: In vivo activity was evaluated in both male and female CF1 mice. Comparison of efficacy against blood-stage P. yoelii demonstrated consistent lead compound activity across both sexes, with ED50 values of 0.35 mg/kg/d for females and 0.33 mg/kg/d for males (Supplementary Fig.1). |

• Liver-Stage Efficacy: These specific assays utilized female Albino C57BL/6 mice.
• Allocation: For all in vivo studies, mice were randomly assigned to treatment and control groups after confirming baseline health and weight.
• Source Data: All findings are disaggregated by sex where applicable in the source data and supplementary files to ensure transparency in biological outcomes.

Field-collected samples

N/A

Ethics oversight

• In vivo blood-stage efficacy studies (VAPORHCS)
o Approving Bodies: VA Portland Institutional Animal Care and Use Committee (IACUC) and the U.S. Army Medical Research and Development Command (USAMRDC) Animal Care and Use Review Office (ACURO).
o Protocol Numbers: Local animal protocol #6067-24 (IRBNet ID #1673475-13) and ACURO protocol #PR210491.e001.
o Approval Dates: Triennial IACUC renewal was granted effective December 18, 2024. The most recent ACURO renewal approval was granted on February 26, 2025.
o Compliance: All research was conducted in an AAALAC International-accredited facility in strict accordance with the Guide for the Care and Use of Laboratory Animals (NRC Publication, Eighth edition), the Animal Welfare Act, and all relevant federal ethical regulations.
• In vivo liver-stage efficacy and PK studies (WRAIR)
o Approving Body: WRAIR/Naval Medical Research Command (NMRC) Institutional Animal Care and Use Committee (IACUC) and the U.S. Army Medical Research and Development Command (USAMRDC) Animal Care and Use Review Office (ACURO).
o Oversight and Approval Dates: Formal administrative oversight and approval were granted by the USAMRDC ACURO on March 27, 2024 (following WRAIR/NMRC IACUC approval.
o Compliance: Research was conducted in an AAALAC International-accredited facility in strict accordance with the Guide for the Care and Use of Laboratory Animals (NRC Publication, eighth edition), Department of Defense (DoD) regulations, and the Animal Welfare Act.
o Protocol Availability: In compliance with DoD and institutional security policies, specific internal protocol numbers are maintained by the WRAIR Office of Animal Care and Compliance and are available for independent verification by the Editorial Office upon request.
• In vivo toxicology and toxicokinetic (TK) studies (SRI International)
o Approving Body: SRI International Institutional Animal Care and Use Committee (IACUC) and the U.S. Army Medical Research and Development Command (USAMRDC) Animal Care and Use Review Office (ACURO).
o Protocol Numbers: SRI IACUC Protocol #02006 and ACURO Protocol #PR210491.e002.
o Approval Date: Formal oversight and approval were granted by ACURO on September 21, 2023.
o Compliance: All studies were conducted in an AAALAC-accredited facility in strict accordance with the Guide for the Care and Use of Laboratory Animals and the U.S. Department of Agriculture (USDA) Animal Welfare Act.

Note that full information on the approval of the study protocol must also be provided in the manuscript.

# Plants

Seed stocks

N/A

Novel plant genotypes

N/A

Authentication

N/A
